# An Etiologic Profile of Anemia in 405 Geriatric Patients

**DOI:** 10.1155/2014/932486

**Published:** 2014-02-23

**Authors:** Tabea Geisel, Julia Martin, Bettina Schulze, Roland Schaefer, Matthias Bach, Garth Virgin, Jürgen Stein

**Affiliations:** ^1^Crohn Colitis Center Rhein-Main, 60594 Frankfurt/Main, Germany; ^2^Institute of Nutritional Science, University of Giessen, 35392 Giessen, Germany; ^3^St. Elisabethen Krankenhaus, 60487 Frankfurt/Main, Germany; ^4^Krankenhaus Sachsenhausen, Teaching Hospital of the J. W. von Goethe University Frankfurt/Main, 60594 Frankfurt/Main, Germany; ^5^Vifor Pharma Deutschland GmbH, 81379 Munich, Germany; ^6^Department of Gastroenterology and Nutritional Medicine, Krankenhaus Sachsenhausen, Teaching Hospital of the J. W. von Goethe University Frankfurt/Main, 60594 Frankfurt/Main, Germany

## Abstract

*Background*. Anemia is a common condition in the elderly and a significant risk factor for increased morbidity and mortality, reducing not only functional capacity and mobility but also quality of life. Currently, few data are available regarding anemia in hospitalized geriatric patients. Our retrospective study investigated epidemiology and causes of anemia in 405 hospitalized geriatric patients. *Methods*. Data analysis was performed using laboratory parameters determined during routine hospital admission procedures (hemoglobin, ferritin, transferrin saturation, C-reactive protein, vitamin B12, folic acid, and creatinine) in addition to medical history and demographics. *Results*. Anemia affected approximately two-thirds of subjects. Of 386 patients with recorded hemoglobin values, 66.3% were anemic according to WHO criteria, mostly (85.1%) in a mild form. Anemia was primarily due to iron deficiency (65%), frequently due to underlying chronic infection (62.1%), or of mixed etiology involving a combination of chronic disease and iron deficiency, with absolute iron deficiency playing a comparatively minor role. *Conclusion*. Greater awareness of anemia in the elderly is warranted due to its high prevalence and negative effect on outcomes, hospitalization duration, and mortality. Geriatric patients should be routinely screened for anemia and etiological causes of anemia individually assessed to allow timely initiation of appropriate therapy.

## 1. Introduction

Iron deficiency is the most prevalent nutritional deficiency worldwide. This metal ion is an essential element in a variety of physiological processes in human beings, including the production of energy in the brain. Iron is also an enzymatic cofactor in the synthesis of neurotransmitters and myelin and is well known for being especially important as a means of oxygen transportation [1]. The main consequence of iron deficiency is anemia, a common condition and significant problem in the older population. However, many physicians continue to neglect the significance of anemia as a serious clinical condition in the elderly [2]. While decreased hemoglobin levels were previously largely considered a normal consequence of aging, there is now evidence that anemia is associated with an increased risk for morbidity and mortality [3, 4]. According to hemoglobin (Hb) cut-off levels defined by the World Health Organization (WHO) (<12 g/dL for females, <13 g/dL for males) [5], anemia is present in 10% of women and 11% of men over the age of 65, increasing to 20% of women and 26% of men over 85 [6]. An even higher prevalence is seen in hospitalized patients, of whom approximately 40–50% have been found to be anemic [7]. The primary consequences of anemia, even mild anemia in which hemoglobin values are only marginally reduced (>9.5 g/dL), are the impairment of functional capacities and a reduced quality of life [8–10]. Furthermore, in elderly persons, anemia can impair physical performance and mobility, thus increasing the risk of falls. An association between anemia in older adults and mortality has been observed in several studies, even in the absence of concomitant illness. In elderly patients, anemia is often overlooked, despite the fact that it has been shown to have potentially serious consequences [2–5, 10].

Data describing the prevalence and causes of anemia in hospitalized geriatric populations are rare and incongruent. Our aim was therefore to determine the epidemiology and etiology of anemia in a hospitalized geriatric population in Germany.

## 2. Methods

### 2.1. Study Design

In this German study, all patients who were admitted between March 2010 and March 2011 to the Geriatric Clinic of the St. Elisabethen Krankenhaus in Frankfurt, Germany, and whose medical records were available were included. The data was analyzed retrospectively.

Patients included in the study were aged 65 years or over. The presence of anemia was defined according to criteria issued by the WHO: hemoglobin (Hb) <12 g/dL for females and <13 g/dL for males. Hb values on admission were available for 386 of the 405 patients (95.3%) and three grades of anemia severity were differentiated: severe (Hb < 8 g/dL), moderate (Hb 8 to <9.5 g/dL), and mild (Hb ≥ 9.5 g/dL). In addition, patient data were assessed for routinely determined levels of serum iron (in 92.6% of patients), serum ferritin (95.6%), transferrin saturation (TSAT) (99.0%), vitamin B_12_ (91.9%), folic acid (88.1%), CRP (96.8%), and serum creatinine (99.51%).

#### 2.1.1. Definition of Anemia Classification

*Anemia Associated with Iron Deficiency. *Patients with Hb levels under 12 g/dL (women) and 13 g/dL (men) and a TSAT value <20% were considered to have anemia associated with iron deficiency. Three subcategories were defined.

- Anemia related to absolute iron deficiency (iron deficiency anemia, IDA) was characterized by a decreased serum ferritin level (<30 *μ*g/mL) in combination with low serum CRP levels (≤0.5 mg/dL).
- Anemia caused by inflammation (AI) was defined by high ferritin levels (>100 *μ*g/mL) and increased CRP (≥0.5 mg/dL).
- Patients with ferritin levels between 30 *μ*g/mL and 100 *μ*g/mL and high CRP levels (≥0.5 mg/dL) were classified as having mixed anemia (IDA/AI).

*Anemia due to Factors Other Than Iron Deficiency. *Patients with Hb levels under 12 g/dL (women) and 13 g/dL (men) and a TSAT value ≥ 20% were considered to have anemia caused by factors other than iron deficiency. Four subcategories were defined.

**Figure 1:**
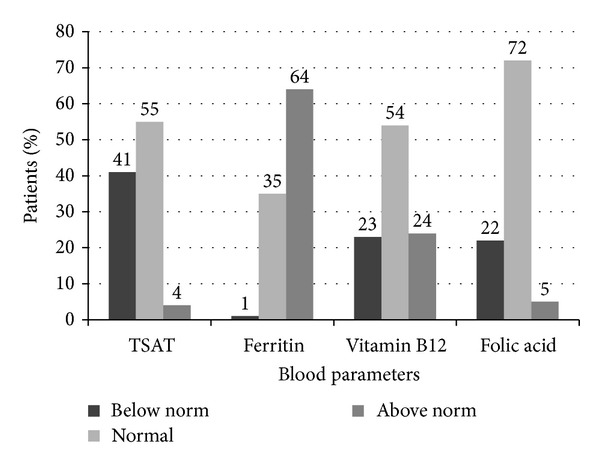
Anemia-related laboratory parameters at time of admission (TSAT = transferrin saturation).

- Anemia secondary to cobalamin deficiency was diagnosed if the serum level was <150 pg/mL.
- Anemia secondary to folic acid deficiency was diagnosed if the serum level was <2 *μ*g/L.
- Anemia of chronic renal insufficiency (CRI) was classified by creatinine values >1.2 mg/dL in females and >1.5 mg/dL in males [11–13].
- Anemia secondary to other etiologies was defined as unexplained anemia (UA) [6]. See Figure 1.

### 2.2. Statistical Analysis

The primary objective of this study was to determine the prevalence of anemia and of different etiological subtypes of anemia in a hospitalized geriatric patient population.

Descriptive statistics were attained through the calculation of arithmetical means, standard deviations, and minimum and maximum values of all data. To test the significance of all categorical variables, the Chi-squared test (Pearson) was performed. Arithmetical means were calculated with *t*-tests for dependent and independent samples, and correlations were determined using the Spearman Rho method. All outcomes with a minimum of *P* < 0.05 were considered significant. Missing values were disregarded in all statistical tests. All statistical analyses were performed using IBM SPSS Statistics 20SPSS statistical software.

## 3. Results

During the period studied, 405 patients (116 men and 289 women) who were admitted to the geriatric clinic had medical records available and were therefore included in the study. The average age was 83.6 ± 6.9 years (range 65–101 years). The patients were divided into three observational groups according to age: 65 to 75 years, 76 to 85 years, and over 85 years of age, representing 12.10%, 36.79%, and 51.11% of the study population, respectively.

The most frequent main causes of hospitalization in this geriatric patient group were fractures (39.4%, *n* = 150), cardiovascular disease (18.4%, *n* = 70), and disturbances of gait and mobility (16.8%, *n* = 64). Other reasons for admission were digestive tract diseases (3.9%, *n* = 15), disorders of the musculoskeletal apparatus (3.7%, *n* = 14), neoplasms (3.7%, *n* = 14), infectious diseases (2.4%, *n* = 9), and injuries (2.4%, *n* = 9), with a further 6.0% (*n* = 23) admitted for other reasons.

Table 1 shows the distribution of these conditions according to specific anemia subtypes. On average, the patients had eight additional diagnoses concurrent to the primary diagnosis, and mean duration of stay in the clinic of all study subjects was 22 days. The main demographic characteristics of the patients are summarized in Table 2.

Of those hospitalized patients whose Hb values at admission were available (*n* = 386), 66.3% (74.8% of men and 62.9% of women) were anemic. There was no correlation between age and Hb level. While only four patients (1.5%) were found to be severely anemic, 37 (13.5%) had moderate anemia and the remaining 85.1% were categorized as having mild anemia.

The total number of patients diagnosed with anemia was 237, of whom 154 (65.0%) were defined as having iron deficiency anemia, with TSAT values <20%. Absolute IDA was found in only 7 (4.6%) of these patients, while 33 (21.4%) had a combination of IDA and AI. The majority of patients with IDA (*n* = 95, 61.7%) were diagnosed with AI, indicated by high CRP and ferritin levels.

Decreased levels of vitamin B_12_ or folic acid were determined as the cause of anemia in 30 patients (5.9% and 6.8%, resp.). In a further 46 (19.4%) study subjects, anemia was found to be the result of chronic renal insufficiency. The remaining patients fell into none of these categories and were therefore classified as having “unexplained anemia.”

The mean serum ferritin level of 315.7 *μ*g/L fell within the normal reference range. Serum ferritin values were, however, increased in the majority of study subjects (64%), while 35% were found to have normal serum ferritin levels.

## 4. Discussion

Anemia is a common condition in the elderly, especially in hospitalized geriatric patients, and is known to be associated with increased morbidity and mortality. The present study was specifically aimed at investigating the epidemiology and etiology of anemia in a hospitalized geriatric population. Blood samples were retrospectively analyzed for the purpose of the study.

The most striking conclusion drawn is the high prevalence of anemia across the board in elderly patients admitted to hospital owing to a wide range of different disorders. Two-thirds of the patients studied were found to be anemic on admission. Although one other study, also focusing on geriatric inpatients, has shown a similar prevalence of anemia [7], most research involving elderly subjects has found the prevalence to be lower [6, 10, 14]. This discrepancy may be accountable to the fact that, in contrast to the current study with its population of hospitalized elderly patients, most other studies have examined ambulant patients or older people in the community [15, 16]. Geriatric persons with health problems severe enough to result in hospital admission are more likely than the geriatric population as a whole to suffer from acute infection and also to have an increased risk of blood loss due to surgery. Thus, hospitalized patients are at a higher risk of developing anemia [17]. Anemia was found in most cases to be mild, with an Hb level > 10 g/dL, in accordance with previously published results [6, 18]. However, even mild anemia is frequently associated with negative outcomes with regard to mortality and morbidity in the elderly [2, 19] and should therefore not be accepted as a normal physiological response to the aging process. Furthermore, the etiological origins of anemia must be determined in all cases in order to facilitate the choice and implementation of effective therapy. It might be seen as a limitation of the present study that reasons for hospital admission were not taken into account. However, we deliberately chose to include all geriatric patients admitted to our clinic, independent of grounds for hospitalization, in order to gain a broader perspective on the prevalence and causes of anemia in elderly patients.

No statistically significant correlation was detected between patients' age and Hb values. This is not in keeping with results of previous research, which have suggested an age-related decrease in Hb levels [6]. Again, this may relate to the specific elderly population included, since all study subjects had serious health issues (and thus, presumably, an increased risk of anemia), whereas in the geriatric population as a whole, the prevalence of serious illness increases with age. Thus, in terms of general state of health, the older the hospitalized patients are, the more representative they can be considered to be of the general population in that age group. As a consequence, what might be considered an innate “bias” of our population towards seriously ill patients (in comparison to studies involving nonhospitalized geriatric persons) is not independent of age, but probably more pronounced in the context of the younger geriatric population.

Determination of the underlying cause of anemia in geriatric persons is complicated by comorbidity and polypharmacy, which are particularly common among the elderly [5]. This must also be taken into account when classifying and comparing the results.

Nonetheless, anemia in the elderly can generally be categorized into four major types: anemia related to nutrient deficiencies (iron, cobalamin, and folic acid), anemia related to chronic inflammation, anemia due to renal insufficiency, and unexplained anemia [6].

Of 237 patients considered to be anemic, 154 (65.0%) had TSAT values <20% and were therefore diagnosed with iron deficiency anemia. In terms of the classification of anemia, the most common etiological subtypes were anemia of inflammation or a mixed form resulting from AI and IDA. Only a few studies have investigated the etiologic profile of anemia in hospitalized patients in the age range from 65 to 101 years [7, 14]. Comparing our results to previous studies is complicated by differences in anemia classification. However, inflammation seems to be the predominant cause of anemia in the observed population, with nutritive factors playing only a limited role [14]. In elderly persons, the causes of anemia vary depending on their clinical setting. AI and IDA are, however, the most common forms of anemia both in community-dwelling and in hospitalized geriatric patients [14]. In our study, IDA was less prevalent than expected in light of results obtained in prior studies.

Only 4.6% of our study subjects with iron deficiency-associated anemia were found to have absolute iron deficiency anemia (IDA), in comparison to 17% in previous reports [6, 20]. A possible explanation for this discrepancy might be the higher mean age (83.6 years) of our patients compared to populations of similar studies, whose mean ages were between 77 and 80 years [13, 21]. Furthermore, we had a high number of comorbidities in our study population. A recent study with a comparable prevalence of comorbidities disclosed an IDA rate of 31% [7].

Sixty-two percent of patients with iron deficiency-related anemia were diagnosed with AI. Petrosyan et al. reported a similar prevalence rate (60%) of AI in a comparable study population [7]. Prevalence rates in community-dwelling elderly persons, however, may be considerably lower. For example, the NHANES (National Health and Nutrition Examination Survey) III demonstrated a prevalence of 24% for this type of anemia in a community-dwelling elderly population [6]. Considerable differences in prevalence may be accountable to the setting (community-dwelling elderly, nursing home residents, or hospital patients), variations in mean age of the study population, and the resultant respective variations in the number of comorbidities and chronic conditions. While AI predominantly occurs as a consequence of chronic or long-term illness or infection, it is also associated with malignancy and inflammatory disorders. These are conditions whose prevalence increases with advancing age and which are more likely to be encountered in a hospitalized setting. Since fractures were the most frequent main cause of hospitalization in our study population, these patients came into the rehabilitation ward. Since an increase in inflammation parameters (CRP and ferritin) is to be expected under these circumstances, this represents an additional explanation for the high prevalence of AI in our study [22]. Studies of noninstitutionalized older persons have demonstrated higher and lower prevalences of IDA and AI, respectively [21, 23].

In our study, anemia of mixed etiology resulting from iron deficiency and chronic inflammation was also analyzed. Thirty-three patients (21.43%) were found to have combined IDA and AI.

Anemia due to deficiencies of vitamin B_12_ or folic acid was found in 5.91% and 6.75% of patients, respectively. Other studies showed higher prevalence rates of 10–20% for cobalamin and 21% for folic acid deficiency [7, 24, 25]. Guralnik et al. determined anemia secondary to folic acid or cobalamin deficiency in 14% of their elderly population [6]. However, comparison of these results is of limited value, as different diagnostic criteria were used.

Anemia of chronic renal insufficiency (CRI) was defined by creatinine values >1.2 mg/dL in females and >1.5 mg/dL in males. Forty-seven (19.41%) of the elderly patients assessed were found to have CRI-related anemia. While previous studies have reported a prevalence of 8–17.5% for anemia resulting from renal insufficiency, most of these studies used a glomerular filtration rate (GFR) of <30 mL/min as the defining criterion for chronic kidney disease [7, 26, 27]. While GFR is indeed considered a better parameter for the diagnosis of chronic renal insufficiency, the present study, due to its retrospective design, was only able to assess chronic renal illness on the basis of the available creatinine values. The results of the studies are therefore not directly comparable.

The patients that fell into none of the given etiological subgroup categories were therefore classified as having unexplained anemia. Possible underlying mechanisms for unexplained anemia include physiological changes such as higher circulating levels of proinflammatory cytokines, myelodysplasia, decreased androgen levels, and a decrease in the proliferative capacity of bone marrow stem cells [6, 28].

Our study has strengths and limitations. The most limiting factor is the retrospective design of the study. Consequently, only those laboratory parameters which were collected as clinical routine on admission were available to be assessed.

Important strengths of the study are the large study population (*n* = 405), the wide spectrum of reasons for admission and of underlying disease or condition, and the use of a variety of different iron and inflammation parameters as assessment criteria for the classification of different causes of anemia.

Conclusive evidence from a large number of studies has confirmed that adequate treatment of iron deficiency significantly improves rates of mortality and morbidity in patients suffering from a wide range of conditions, including chronic heart failure [29, 30], coronary heart disease [31], chronic kidney disease [32, 33], cancer [34, 35], and rheumatoid arthritis [36, 37]. Nevertheless, screening and treatment of ID continue to be widely neglected in the routine management of geriatric patients. There is clearly a need for greater awareness of the high prevalence of anemia in the elderly and of its significance in terms of poorer outcomes, prolonged hospital stays, and increased mortality. Our study underlines the importance of routine screening and individual assessment of the etiological causes of anemia in geriatric patients, allowing the timely initiation of optimal and appropriate therapy. In addition, the perioperative administration of intravenous iron is advisable in order to reduce anemia-related complications and minimize transfusion requirements.

Rather than relying on a single biomarker, screening should include a range of parameters including TSAT, serum ferritin, and CRP. A new generation of intravenous iron preparations allows rapid single-session doses of up to 1,000 mg, thus offering an excellent option for effective treatment and prevention of iron deficiency in all patients, including the elderly [38]. Dosage can be calculated using standard calculation methods such as the Ganzoni formula.

## Figures and Tables

**Table 1:** Anemia subtypes according to main reason for hospitalization.

	All patients *n* = 368 *n* (%*)	All anemic patients *n* = 241 *n* (%^∗1^)	IDA *n* (%^∗1^)	IDA/AI *n* (%^∗1^)	AI *n* (%^∗1^)	B_12_/folic acid deficiency *n* (%^∗1^)	Renal anemia *n* (%^∗1^)	UA *n* (%^∗1^)
Fractures	150 (40.76)	110 (43.90)	0 (0)	9 (8.18)	31 (28.18)	8 (7.27)	10 (9.09)	52 (47.27)
Cardiovascular disease	70 (19.02)	40 (57.14)	3 (7.50)	6 (15.00)	15 (37.50)	3 (7.50)	7 (17.50)	6 (15.00)
Disturbance of gait and mobility	64 (17.39)	26 (40.63)	1 (3.85)	5 (19.23)	3 (11.54)	2 (7.69)	5 (19.23)	10 (38.46)
Digestive tract diseases	15 (4.08)	13 (86.67)	1 (7.69)	2 (15.38)	4 (30.77)	2 (15.38)	4 (30.77)	0 (0)
Disorders of the musculoskeletal apparatus	14 (3.80)	13 (92.86)	0 (0)	1 (7.69)	3 (23.08)	3 (23.08)	6 (46.15)	0 (0)
Neoplasma	14 (3.80)	10 (71.43)	0 (0)	1 (10)	6 (60.00)	2 (20)	1 (10)	0 (0)
Infectious diseases	9 (2.45)	7 (77.78)	1 (14.29)	2 (28.57)	2 (28.57)	1 (14.29)	1 (14.92)	0 (0)
Injuries	9 (2.45)	4 (44.44)	0 (0)	2 (50.00)	0 (0)	1 (25.00)	1 (25)	0 (0)
Other reasons	23 (6.25)	18 (78.26)	1 (5.56)	3 (16.67)	4 (22.22)	2 (11.11)	4 (22.22)	4 (22.22)
		241 (100)	7 (2.90^∗2^)	31 (12.86^∗2^)	68 (28.22^∗2^)	24 (9.96^∗2^)	39 (16.18^∗2^)	72 (29.88^∗2^)

*Relating to all hospitalized patients.

^∗1^Relating to all anemic patients with this diagnosis.

^∗2^Relating to all anemic patients.

**Table 2:** Iron parameters in all patients subdivided into age groups.

	Number of patients		Mean ± SD		Median		Min.		Max.	
	Female	Male	Female	Male	Female	Male	Female	Male	Female	Male
Hb (g/dL)										
65–75 years	33	21	11.67 ± 3.01	11.84 ± 2.05	11.5	11.6	9	8.5	19.5	16.5
76–85 years	97	59	11.58 ± 2.24	11.91 ± 1.81	11.6	11.4	8.1	9.1	19.7	16.2
>85 years	149	29	11.30 ± 2.0	11.94 ± 1.97	11.4	12.4	8.1	8.9	16.9	16.2
Serum iron (mg/dL)										
65–75 years	32	19	3.53 ± 1.93	5.05 ± 3.08	3	5	1	1	8	9
76–85 years	97	57	4.15 ± 2.36	4.26 ± 2.117	4	5	1	1	9	8
>85 years	142	27	4.7 ± 2.38	3.85 ± 2.37	5	4	1	1	9	8
Serum ferritin (*µ*g/L)										
65–75 years	35	22	184.14 ± 206.62	371.36 ± 232.83	116	314	29	41	1131	905
76–85 years	94	58	262.38 ± 263.06	336.71 ± 284.13	201.5	253	18	56	2026	1435
>85 years	149	27	262.38 ± 263.06	336.56 ± 291.14	212	216	7	38	1352	1250
TSAT (%)										
65–75 years	36	22	18.64 ± 12.35	18.18 ± 9.39	15	18.5	4	4	60	35
76–85 years	100	61	20.44 ± 12.39	21.44 ± 11.61	18	20	3	6	87	67
>85 years	153	30	19.62 ± 10.03	20.97 ± 14.41	17	18	5	6	52	79
CRP (mg/dL)										
65–75 years	35	22	4.77 ± 6.29	1.47 ± 1.85	2.3	0.65	0.2	0.2	28.7	7.4
76–85 years	127	82	4.77 ± 6.29	2.19 ± 4.02	1.7	0.95	0	0.2	28.7	30
>85 years	151	28	3.88 ± 6.08	1.7 ± 0.7	1.9	0.7	0	0	49	9.8

## Abbreviations

AI: Anemia of inflammation
CRI: Chronic renal insufficiency
CRP: C-reactive protein
GFR: Glomerular filtration rate
IDA: Iron deficiency anemia
NHANES: National Health and Nutrition Examination Survey
TSAT: Transferrin saturation
UA: Unexplained anemia
WHO: World Health Organization.
